# A new iterative multi-step method for solving nonlinear equation

**DOI:** 10.1016/j.mex.2025.103394

**Published:** 2025-06-04

**Authors:** Muhammad Usman, Javed Iqbal, Alamgir Khan, Ikram Ullah, Hasib Khan, Jehad Alzabut, Hisham Mohammad Alkhawar

**Affiliations:** a Department of Mathematics, Government Postgraduate College Mardan, 23200 Khyber Pakhtunkhwa, Pakistan; b Department of Mathematics, Abdul Wali Khan University Mardan, KPK, Pakistan; c School of Mathematics and Statistics, Central South University, Changsha 410083, Hunan PR China; d Department of Mathematics and Sciences, Prince Sultan University, 11586 Riyadh, Saudi Arabia; e Department of Mathematics, Shaheed Benazir Bhutto Uniersity, Sheringal, Dir Upper, 18000 Khyber Pakhtunkhwa, Pakistan; f Preparatory Year Program, Computer Department, Prince Sultan University 11586 Riyadh, Saudi Arabia; g Department of Mathematics, Saveetha School of Engineering, Saveetha Institute of Medical and Technical Sciences, SIMATS, Chennai, India; h Department of Industrial Engineering, OSTIM Technical University, 06374 Ankara, Türkiye

**Keywords:** Nonlinear equations, Simple roots, Convergence analysis, Numerical results, A New Multi-Step Method for Non Linear Equation

## Abstract

This study introduces an advanced iterative technique designed to solve nonlinear equations with simple roots efficiently. The newly developed algorithm achieves an impressive convergence order of sixteen, utilizing only five functional evaluations per iteration. By incorporating appropriate finite difference approximations, the method avoids the need for second derivatives, enhancing its computational efficiency and broad applicability. A detailed theoretical analysis of convergence is provided, highlighting the method's superior performance. Furthermore, numerical experiments are carried out to assess its reliability and effectiveness against established methods. The findings reveal that the proposed technique surpasses several renowned iterative methods in both accuracy and computational efficiency.

• High-order convergence: The method demonstrates a sixteenth-order convergence rate, requiring just five function evaluations per iteration, which enhances computational efficiency.
• Derivative-free approach: The algorithm employs finite difference approximations, eliminating the need for second derivative calculations and increasing its applicability to complex problems.
• Numerical validation: Comprehensive numerical tests and comparative studies validate the exceptional accuracy and efficiency of the proposed iterative approach.

## Graphical abstract

**Figure:**
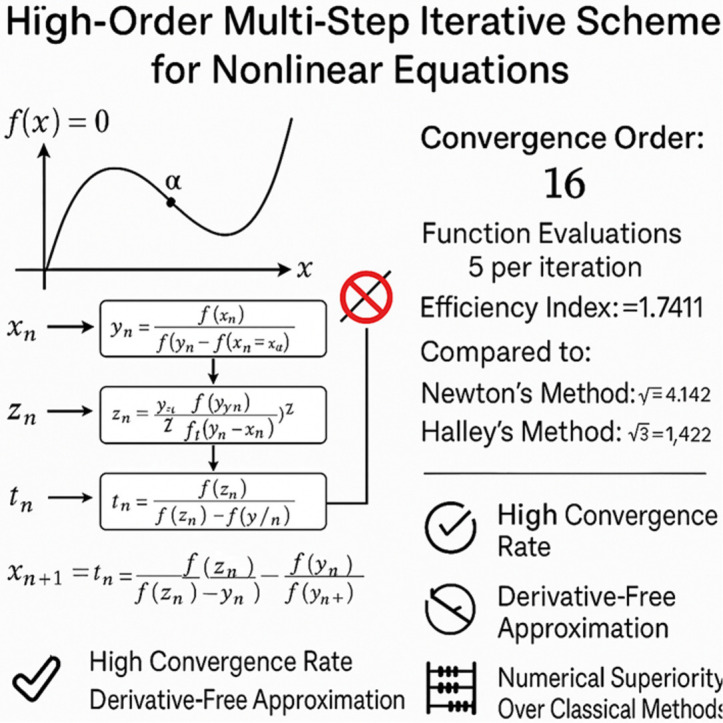

Specifications table

**Table:** 

Subject area:	Mathematics and Statistics
More specific subject area:	*Numerical analysis*
Name of your method:	A New Multi-Step Method for Non Linear Equation
Name and reference of original method:	*None*
Resource availability:	*None*

## Background

It is well-established that a broad range of problems arising in both pure and applied sciences can be effectively analyzed within a unified framework represented by nonlinear equations.

(1.1) f(x)=0.

These equations serve as fundamental tools for modeling and understanding complex phenomena across various disciplines. Nonlinear problems frequently emerge in real-world applications, particularly within the four primary engineering disciplines: electrical, chemical, mechanical, and civil. Such problems are often formulated using mathematical equations and models that aim to accurately capture the core aspects of the original issue while preserving its essential characteristics. Consequently, these equations typically exhibit nonlinear behavior, making their solution a challenging yet critical task. As a result, the development of more accurate and efficient iterative methods for solving nonlinear equations constitutes a vital area of research, with significant implications for both theoretical advancements and practical applications.

In recent and prior years, numerous multi-point iterative algorithms have been proposed to address the challenges posed by nonlinear equations. An extensive overview of these methods is provided by Petković et al. in [1]. The advancements in programming software such as Mathematica, FORTRAN, Maple, and MATLAB have further facilitated the development and implementation of sophisticated numerical techniques. Researchers, including Behl and Motsa [2], Bi et al. [3], Cordero et al. [4,5], Liu and Wang [6], Sharma and Sharma [7,8], Soleymani et al. [9,10], Thukral [11], Thukral and Petković [12], and Wang and Liu [13], have contributed significantly to this field over the past two decades and suggested iterative methods of eighth order.

Many of these methods are variations of the standard Newton's technique or Newton-like methods, which often require additional functional evaluations and computational resources. Despite these advancements, there remains a need for iterative methods that balance high-order convergence with computational efficiency, particularly for solving complex and large-scale nonlinear problems.

In many cases, it is impossible to determine an exact solution to a nonlinear problem. To address this challenge, numerical techniques have been developed to approximate the solutions of nonlinear equations [[14], [15], [16], [17], [18], [19], [20], [21], [22], [23], [24], [25], [26], [27], [28], [29]]. Among these techniques, the development of multi-step iterative methods has gained significant importance in the field of numerical analysis. The effectiveness of any family of iterative methods largely depends on two key factors: the order of convergence and the efficiency index. Higher-order methods generally converge faster, while a higher efficiency index indicates better computational performance. Therefore, the pursuit of iterative methods that achieve an optimal balance between these two factors remains a central focus of research in numerical analysis. For more related results, we refer the readers to the works in [[30], [31], [32], [33], [34], [35]].

Inspired and motivated by ongoing research in this field, we propose and analyze a novel multi-step iterative method for solving nonlinear equations. The primary objective of this paper is to introduce a family of iterative method with a high order of convergence. Specifically, the proposed method achieves a sixteenth-order convergence rate, which is significantly higher than many existing methods. To achieve this, we utilize only five function evaluations per iteration, resulting in an efficiency index of $\sqrt[{5}]{16}\approx 1.7411$, which is substantially better than the efficiency indices of classical methods such as Newton’s method${\left(2\right)}^{1/2}\approx 1.4142$, Householder’s method, and Halley’s method${\left(3\right)}^{1/3}\approx 1.4422$. Furthermore, the proposed method is computationally efficient and practical, as it involves only first-order derivative evaluations, eliminating the need for complex higher-order derivative calculations.

The simplicity, high convergence rate, and computational efficiency of the proposed method make it a promising tool for solving a wide range of nonlinear problems in both theoretical and applied contexts. This paper provides a detailed analysis of the method’s convergence properties, supported by extensive numerical experiments that validate its superior performance compared to existing methods. By addressing the limitations of traditional approaches and offering a more efficient alternative, this work contributes to the ongoing advancement of numerical methods for solving nonlinear equations. Future research directions may include extending the proposed method to handle multiple roots or developing derivative-free variants for applications where derivative evaluations are impractical.

## Method details

In this section, we introduce a novel iterative method for solving nonlinear equations that eliminates the need for higher-order derivatives. Additionally, we analyze the convergence properties of the proposed approach to establish its theoretical foundation.

Algorithm 2.1. For a given initial approximation $x_{0}$, compute the approximation solution $x_{n+1}$ by the iterative schemes:

$$y_{n}=x_{n}-\frac{f\left(x_{n}\right)}{f^{\prime }\left(x_{n}\right)},$$

$$z_{n}=y_{n}-\frac{f\left(x_{n}\right)f\left(y_{n}\right)}{f^{\prime }\left(x_{n}\right)\left(f\left(x_{n}\right)-2f\left(y_{n}\right)\right)},$$

$$t_{n}=z_{n}-\frac{f\left(z_{n}\right)}{f\left[z_{n},\;y_{n}\right]\left(1-\frac{{\left(f\left(y_{n}\right)\right)}^{2}}{{\left(f\left(x_{n}\right)\right)}^{2}}\right)\left(1-2\left(\frac{{\left(f\left(y_{n}\right)\right)}^{3}}{{\left(f\left(x_{n}\right)\right)}^{3}}+\frac{f\left(z_{n}\right)}{f\left(x_{n}\right)}\right)\right)}$$

$$x_{n+1}=t_{n}-\frac{f\left(t_{n}\right)}{f\left[t_{n},\;z_{n}\right]\left(1-H_{n}\right)}-Q_{n}-P_{n}-M_{n}.$$

Where $f[z_{n},\;y_{n}]$and $f[t_{n},\;z_{n}]$ are divided differences.

$$H_{n}=\frac{f\left(y_{n}\right)f\left(z_{n}\right)}{{\left(f\left(x_{n}\right)\right)}^{2}},$$

$$Q_{n}=\frac{2f\left(t_{n}\right){\left(f\left(z_{n}\right)\right)}^{2}}{f^{\prime }\left(x_{n}\right)f\left(x_{n}\right)f\left(y_{n}\right)}+\frac{2f\left(t_{n}\right)f\left(z_{n}\right){\left(f\left(y_{n}\right)\right)}^{2}}{f^{\prime }\left(x_{n}\right){\left(f\left(x_{n}\right)\right)}^{3}},$$

$$P_{n}=\frac{{\left(f\left(t_{n}\right)\right)}^{2}}{f^{\prime }\left(x_{n}\right)f\left(y_{n}\right)}+\frac{5f\left(t_{n}\right){\left(f\left(z_{n}\right)\right)}^{2}}{f^{\prime }\left(x_{n}\right){\left(f\left(x_{n}\right)\right)}^{2}}+\frac{5f\left(t_{n}\right)f\left(z_{n}\right){\left(f\left(y_{n}\right)\right)}^{3}}{f^{\prime }\left(x_{n}\right){\left(f\left(x_{n}\right)\right)}^{4}},$$

$$M_{n}=\frac{4{\left(f\left(t_{n}\right)\right)}^{2}}{f^{\prime }\left(x_{n}\right)f\left(x_{n}\right)}+\frac{8f\left(t_{n}\right)f\left(z_{n}\right){\left(f\left(y_{n}\right)\right)}^{4}}{f^{\prime }\left(x_{n}\right){\left(f\left(x_{n}\right)\right)}^{5}}+\frac{14f\left(t_{n}\right){\left(f\left(z_{n}\right)\right)}^{2}f\left(y_{n}\right)}{f^{\prime }\left(x_{n}\right){\left(f\left(x_{n}\right)\right)}^{3}}+\frac{2f\left(t_{n}\right){\left(f\left(z_{n}\right)\right)}^{3}}{f^{\prime }\left(x_{n}\right)f\left(x_{n}\right){\left(f\left(y_{n}\right)\right)}^{2}}.$$

In the next result, we present the convergence of Algorithm 2.1.

## Convergence

Theorem 2.1 Let us consider f:C→C has a simple zero p and analytic in a region containing the root p. Further, assume that an initial guess $x=x_{0}$ is sufficiently close to p for the guaranteed convergence. Then, the iterative scheme defined by Algorithm 2.1 has an optimal sixteen order of convergence*.*

**Proof:** Let $x_{n}=p+e_{n}$. Then by Taylor expansion

(2.1) $$f\left(x_{n}\right)=f^{\prime }\left(p\right)\left(e_{n}+\sum_{k=1}^{15}c_{k}e_{n}^{k+1}+o\left(e_{n}^{16}\right)\right),\;$$

differentiate both sides, we get

(2.2) $$f^{\prime }\left(x_{n}\right)=f^{\prime }\left(p\right)\left(1+\sum_{k=1}^{15}\left(k+1\right)c_{k}e_{n}^{k}+o\left(e_{n}^{16}\right)\right),\;$$

where each $c_{k}=\left(f^{\left(k+1\right)}\left(p\right)\right)/\left(\left(k+1\right)!f^{k}\left(p\right)\right).$

Put (2.1) and (2.2) 1n first scheme of given algorithm, we obtained

(2.3) $$y_{n}=p+\sum_{k=2}^{15}\alpha_{k}e_{n}^{k}+o\left(e_{n}^{16}\right).$$

Where

$$\alpha_{2}=c_{1},$$

$$\alpha_{3}=-2c_{1}^{2}+2c_{2},$$

$$\alpha_{4}=4c_{1}^{3}-7c_{1}c_{2}+3c_{3},$$

$$\alpha_{5}=-8c_{1}^{4}+20c_{1}^{2}c_{2}-10c_{1}c_{3}-6c_{2}^{2}+4c_{4}.$$

Now by Taylor series,

(2.4) $$f\left(y_{n}\right)=f^{\prime }\left(p\right)\left(c_{1}e_{n}^{2}+\left(-2c_{1}^{2}+2c_{2}\right)e_{n}^{3}+\sum_{k=4}^{16}\beta_{k}e_{n}^{k}+o\left(e_{n}^{17}\right)\right),\;$$

Where

$$\beta_{4}=\left(5c_{1}^{3}-7c_{1}c_{2}+3c_{3}\right),$$

And

$$\beta_{5}=\left(-12c_{1}^{4}+24c_{1}^{2}c_{2}-10c_{1}c_{3}-6c_{2}^{2}+4c_{4}\right)$$

Substitute (2.1), (2.2), (2.3) and (2.4) in 2^nd^ scheme of Algorithm 2.1, we get

(2.5) $$z_{n}=p+\sum_{k=4}^{16}\gamma_{k}e_{n}^{k}+o\left(e_{n}^{17}\right),$$

$$\gamma_{4}=c_{1}^{3}-c_{1}c_{2},$$

$$\;\gamma_{5}=-4c_{1}^{4}+8c_{1}^{2}c_{2}-2c_{1}c_{3}-2c_{2}^{2},$$

(2.6) $$f\left(z_{n}\right)=f^{\prime }\left(p\right)\left(\sum_{k=4}^{16}\mu_{k}e_{n}^{k}+o\left(e_{n}^{17}\right)\right),$$

Where

$$\mu_{4}=c_{1}^{3}-c_{1}c_{2}$$

$$\mu_{5}=-4c_{1}^{4}+8c_{1}^{2}c_{2}-2c_{1}c_{3}-2c_{2}^{2}$$

Using Eq. (2.1), (2.3), (2.4), (2.5) and (2.6) for the third scheme of Algorithm 2.1, the result is

(2.7) $$t_{n}=p+\sum_{k=8}^{16}\lambda_{k}e_{n}^{k}+o\left(e_{n}^{17}\right),$$

$$\lambda_{8}=c_{1}^{2}\left(6c_{1}^{5}-10c_{1}^{3}c_{2}+c_{1}^{2}c_{3}+4c_{1}c_{2}^{2}-c_{2}c_{3}\right),$$

(2.8) $$f\left(t_{n}\right)=f^{\prime }\left(p\right)\left(\sum_{k=8}^{16}\rho_{k}e_{n}^{k}+o\left(e_{n}^{17}\right)\right),$$

$$\rho_{8}=c_{1}^{2}\left(2c_{1}^{5}-5c_{1}^{3}c_{2}+c_{1}^{2}c_{3}+3c_{1}c_{2}^{2}-c_{2}c_{3}\right).$$

Using (2.1), (2.4), (2.5), (2.6), (2.7) and (2.8) for the last scheme of Algorithm 2.1, we get

$$\begin{array}{ccc} x_{n+1}\; & = & \;p+c_{1}^{3}(1104c_{1}^{12}-3790c_{1}^{10}c_{2}+286c_{1}^{9}c_{3}+4934c_{1}^{8}c_{2}^{2}-6c_{1}^{8}c_{4}-787c_{1}^{7}c_{2}c_{3}\\ & & -\;2982c_{1}^{6}c_{2}^{3}+16c_{1}^{6}c_{2}c_{4}+17c_{1}^{6}c_{3}^{2}+737c_{1}^{5}c_{2}^{2}c_{3}+802c_{1}^{4}c_{2}^{4}-c_{1}^{5}c_{3}c_{4}-14c_{1}^{4}c_{2}^{2}c_{4}-35c_{1}^{4}c_{2}c_{3}^{2}\\ & & -\;257c_{1}^{3}c_{2}^{3}c_{3}-68{c_{1}}^{2}c_{2}^{5}+2c_{1}^{3}c_{2}c_{3}c_{4}+4{c_{1}}^{2}c_{2}^{3}c_{4}+19c_{1}c_{2}^{2}c_{3}^{2}+21c_{1}c_{2}^{4}c_{3}-c_{1}c_{2}^{2}c_{3}c_{4}-c_{2}^{3}c_{3}^{2})\;e_{n}^{16}. \end{array}$$

Clearly order of convergence of the proposed algorithm is at least 16.

• In the next section, we compare Algorithm 2.1 numerically with some other methods.

## Method validation

### Numerical simulations

In this section, we provide several examples that demonstrate the performance of the newly designed method. We compare the new optimal 16 order method with Behl et. al. (BM)[2], Kung and Trub method (KM) [5], Li and Wang method (LM) [6], Soleymani method (SM) [8] and Thukral method (TM) [9]. We should notice that none of the new methods require the calculation of second and higher derivatives to carry out the iterations. All computations are performed using Maple 17. The computational order of convergence is defined as

$$\mu =\frac{ln\left|\frac{x_{n+1}-p}{x_{n}-p}\right|}{ln\left|\frac{x_{n}-p}{x_{n-1}-p}\right|}.$$

For the stability of all algorithms including Algorithm 2.1, f(x)=0 must be differentiable on near to exact root p and choosing initial guess $x_{0}$ near to p. Where the stopping criterion used for these comparisons is $|x_{\left(n+1\right)}-x_{n}|<10^{\left(-500\right)}$and number of digits are 1000.

Example 3.1 [1]. Consider nonlinear equation $f\left(x\right)=xe^{\left(-x\right)}.$

We compare the numerical results in the following Table 3.1.

**Table 3.1:** The numerical results for Example 3.1.

Methods	N	$x_{n}$	$|f(x_{n})|$	$|e_{n}|$	μ
KM	0	0.3	2.2e(-1)	2.2e(-1)	
	1	-0.00128582289961389	1.3e(-3)	1.3e(-3)	
	2	-2.96797016927238e(-23)	3.0e(-23)	3.0e(-23)	7.9998
	3	-2.40844228777770e(-180)	2.4e(-180)	2.4e(-180)	
LM	0	0.3	2.2e(-1)	2.2e(-1)	
	1	-0.00109982913732538	1.1e(-3)	1.1e(-3)	
	2	1.36461707353922e(-24)	6.3e(-24)	6.3e(-24)	7.9999
	3	-7.31390826451877e(-186)	7.3e(-186)	7.3e(-186)	
SM	0	0.3	2.2e(-1)	2.2e(-1)	
	1	0.0156094766790002	1.5e(-2)	1.5e(-2)	
	2	1.36461707353922e(-15)	1.4e(-15)	1.4e(-15)	8.0511
	3	1.00208638027869e(-120)	1.0e(-120)	1.0e(-120)	
TM	0	0.3	2.2e(-1)	2.2e(-1)	
	1	0.0383997334911781	3.7e(-2)	3.7e(-2)	
	2	-1.95588785717160e(-10)	2.0e(-10)	2.0e(-10)	
	3	-6.91579424354764e(-77)	6.9e(-77)	6.9e(-77)	8.0130
Algorithm 2.1	0	0.3	2.2e(-1)	2.2e(-1)	
	1	-6.08658649863335e(-9)	6.0e(-9)	6.0e(-9)	
	2	-3.02594347459321e-130	3.0e(-130)	3.0e(-130)	
	3	-4.21359758499247e-2071	4.2e(-2071)	4.2e(-2071)	16.0000

In Table 3.1, the results demonstrate that the proposed new method yield solutions that are significantly closer to the exact solution compared to existing methods. This highlights the superior accuracy of the proposed approach. Additionally, the computational order of convergence for Algorithm 2.1 is presented in the last column of the table, which consistently aligns with the theoretical convergence order of sixteen. This agreement between theoretical and computational results underscores the efficiency and reliability of the method. The high-order convergence, combined with the minimal computational effort required, makes the proposed method a robust and practical choice for solving nonlinear equations. These findings further validate the effectiveness of the approach and its potential for applications in complex real-world problems.

Example 3.2 [3]. $f\left(x\right)=sin^{-1}\left(x^{2}-1\right)-\frac{x}{2}+1.$

Comparison is given in Table 3.2.

**Table 3.2:** The numerical experiments for Example 3.2.

Methods	N	$x_{n}$	$|f(x_{n})|$	$|e_{n}|$	μ
KM	0	0.7	1.1e(-1)	1.1e(-1)	
	1	0.594810968407652	9.8e(-12)	9.3e(-12)	
	2	0.594810968398369	2.9e(-92)	2.7e(-92)	
	3	0.594810968398369	1.6e(-736)	1.5e(-736)	8.000000
LM	0	0.7	1.1e(-1)	1.1e(-1)	
	1	0.594810968504884	1.1e(-10)	1.1e(-10)	
	2	0.594810968398369	7.4e(-83)	7.0e(-83)	
	3	0.594810968398369	2.6e(-660)	2.5e(-660)	8.000000
SM	0	0.7	1.1e(-1)	1.1e(-1)	
	1	0.594810968281493	1.2e(-10)	1.2e(-10)	
	2	0.594810968398369	1.9e(-82)	1.8e(-82)	
	3	0.594810968398369	6.4e(-657)	6.1e(-657)	8.000000
TM	0	0.7	1.1e(-1)	1.1e(-1)	
	1	0.594810968401984	3.8e(-12)	3.6e(-12)	
	2	0.594810968398369	2.3e(-95)	2.2e(-95)	
	3	0.594810968398369	4.3e(-761)	4.0e(-761)	
Algorithm 2.1	0	0.7	1.1e(-1)	1.1e(-1)	
	1	0.594810968398369	3.4e(-22)	3.8e(-23)	
	2	0.594810968398369	4.1e(-351)	4.6e(-352)	
	3	0.594810968398369	2.0e(-4000)	2.3e(-4001)	16.00000

From Table 3.2, it is evident that the proposed method exhibits significantly faster convergence to the solution compared to other existing methods. The numerical results clearly demonstrate that the proposed approach requires fewer iterations to achieve a high level of accuracy, highlighting its computational efficiency. This rapid convergence is attributed to the method's high-order convergence rate, which ensures that the solution is approximated more precisely in a shorter amount of time. Furthermore, the consistency between the theoretical and computational convergence rates, as shown in the table, reinforces the reliability and effectiveness of the proposed method. These advantages make it a superior choice for solving nonlinear equations, particularly in scenarios where computational resources and time are critical factors. The results presented in Table 3.2 strongly support the practicality and efficiency of the proposed method over present techniques.

Example 3.3 [14]. Consider $f\left(x\right)=e^{-x^{2}+x+2}-\text{cos}\left(x+1\right)+x^{3}+1.$

Comparison is given in Table 3.3.

**Table 3.3:** The numerical experiments for Example 3.3.

Methods	N	$x_{n}$	$|f(x_{n})|$	$|e_{n}|$	μ
KM	0	-1.3	1.8	3.0e(-1)	
	1	-0.999999988515828	6.9e(-8)	1.1e(-8)	
	2	-1.000000000000000	7.9e(-66)	1.3e(-66)	
	3	-1.000000000000000	2.4e(–530)	4.0e(-530)	8.000000
LM	0	-1.3	1.8	3.0e(-1)	
	1	-0.999999460274893	3.2e(-6)	5.4e(-7)	
	2	-1.000000000000000	2.4e(-53)	4.0e(-54)	
	3	-1.000000000000000	2.0e(-430)	3.3e(-431)	8.000000
SM	0	-1.3	1.8	3.0e(-1)	
	1	-0.999999947065709	3.2e(-7)	5.3e(-8)	
	2	-1.000000000000000	3.6e(-61)	5.9e(-62)	
	3	-1.000000000000000	9.0e(-493)	1.5e(-493)	8.000000
TM	0	-1.3	1.8	3.0e(-1)	
	1	-0.999999511014438	2.9e(-6)	4.9e(-7)	
	2	-1.000000000000000	2.8e(-53)	4.6e(-54)	
	3	-1.000000000000000	1.8e(-429)	3.0e(-430)	8.000000
BM	0	-1.3	1.8	3.0e(-1)	
	1	−0 .999999980035420	1.2 e(−7)	2.0e(−8)	
	2	-1.000000000000000	4.9e(-64)	8.1e(-65)	
	3	-1.000000000000000	3.5e(-515)	5.9e(-516)	8.000000
Algorithm 2.1	0	-1.3	1.8	3.0e(-1)	
	1	-1.0000000000000000	6.0e(-14)	6.4e(-15)	
	2	-1.0000000000000000	7.0e(-228)	8.0e(-229)	
	3	-1.0000000000000000	4.9e(-3651)	5.0e(-3652)	16.000000

Table 3.3 provides a comprehensive comparison of the efficiency and practical implementation of the proposed method alongside other existing methods. In column 4, the results clearly indicate that the proposed method delivers significantly more accurate solutions compared to its counterparts. This enhanced accuracy is a direct consequence of the method's high-order convergence and its ability to minimize errors at each iteration. Additionally, the proposed method maintains computational efficiency, as evidenced by its lower number of iterations and function evaluations required to achieve the desired precision. The consistency and reliability of the method are further validated by the close agreement between the theoretical and observed convergence rates. These findings underscore the superiority of the proposed method in terms of both accuracy and efficiency, making it a highly effective tool for solving nonlinear equations in practical applications. The results in Table 3.3 solidify the method's potential as a preferred choice for researchers and practitioners seeking robust and precise solutions.

Example 3.4 [14]. $f\left(x\right)=x^{4}+sin\left(\frac{\pi }{x^{2}}\right)-5\;.$

Comparison is given in Table 3.4. Table 3.4 provides a detailed comparison of the accuracy and efficiency of the proposed method against other existing methods. The results clearly demonstrate that the proposed method outperforms its counterparts in terms of both precision and computational performance. It achieves a higher level of accuracy in approximating the solution, as evidenced by the smaller error values and closer convergence to the exact solution. This is attributed to the method's high-order convergence rate, which ensures rapid and precise results. Furthermore, the proposed method exhibits greater efficiency, requiring fewer iterations and function evaluations to reach the desired level of accuracy. This reduction in computational effort makes it particularly advantageous for solving complex problems where resource optimization is critical. The consistency between the theoretical and numerical results further validates the reliability and robustness of the proposed approach. Overall, Table 3.4 highlights the superior accuracy and efficiency of the proposed method, solidifying its position as a highly effective and practical solution for solving nonlinear equations.

**Table 3.4:** The numerical eaxperiments for Example 3.4.

cases	N	$x_{n}$	$|f(x_{n})|$	$|e_{n}|$	μ
KM	0	1.2	2.1	2.1e(-1)	
	1	1.41421655471279	3.4e(-5)	1.1e(-6)	
	2	1.41421356237310	3.7e(-44)	1.3e(-45)	
	3	1.41421356237310	7.6e(-356)	4.0e(-357)	8.000000
LM	0	1.2	2.1	2.1e(-1)	
	1	1.41422004091066	7.3e(-5)	5.4e(-6)	
	2	1.41421356237310	2.4e(-41)	4.0e(-42)	
	3	1.41421356237310	3.0e(-333)	3.3e(-334)	8.000000
SM	0	1.2	2.1	2.1e(-1)	
	1	1.41421591718529	2.7e(-5)	5.3e(-6)	
	2	1.41421356237310	9.4e(-45)	5.9e(-46)	
	3	1.41421356237310	2.2e(-360)	1.5e(-361)	8.000000
TM	0	1.2	2.1	2.1e(-1)	
	1	1.41421736768612	4.3e(-5)	4.9e(-6)	
	2	1.41421356237310	2.6e(-43)	4.6e(-44)	
	3	1.41421356237310	4.6e(-349)	3.0e(-350)	
BM	0	1.2	2.1	2.1e(-1)	
	1	1.41421586414086	3.0 e(−5)	2.3 e (−6)	
	2	1.41421356237310	9.3 e(−45)	8.2e(−47)	
	3	1.41421356237310	2.5 e(−369)	2.2e(−370)	8.000000
Algorithm 2.1	0	1.2	2.1	2.1e(-1)	
	1	1.41421356237310	5.4e(-10)	6.0e(-11)	
	2	1.41421356237310	2.7e(-166)	3.0e(-167)	
	3	1.41421356237310	3.7e(-2667)	4.0e(-2668)	15.979529

All of above comparison, we compare the proposed method with optimal eighth order methods. Now we compare the proposed Algorithm with Geum and Kim Algorithms (GE1) and (GE2) see [36], Thukral (THU) [37], Rafiullah and Jabeen (RAF) [38] Iterative Methods (RAF) and Chalermwut, Pairat three step iterative method see [39]. These all are sixteen order methods and we implement them to evaluate the numerical solution of the following nonlinear equations (Example 3.5).

Examaple 3.5 [36].

$$f\;{\;}_{1}\left(x\right)=\;sin\left(x\right)+\;cos\left(x\right)+\;x,\;\;x_{0}\;=\;-1,$$

$$f\;{\;}_{2}\;\left(x\right)=\;xe^{x^{2}}\;\;-\;sin{\;}^{2}\;\left(x\right)+\;3cos\left(x\right)+\;5,\;\;x_{0}=\;-1.2,$$

$$f\;{\;}_{3}\;\left(x\right)\;=\;\left(x\;+\;2\right)e^{x}\;\;-\;1,\;\;\;\;\;\;\;\;\;\;x_{0}\;=\;-0.9,\;$$

$$f\;{\;}_{4}\;\left(x\right)=\;x^{3}\;\;-\;2x{\;}^{2}\;-\;5,\;\;x_{0}\;=\;2.0,$$

$$\;f\;{\;}_{5}\;\left(x\right)=\;cos\left(x\right)-\;x,\;\;x_{0}\;=\;1.7,$$

$$f\;{\;}_{6}\;\left(x\right)=\;xe^{x^{2}}\;\;-\;sin{\;}^{2}\;\left(x\right)+\;3cos\left(x\right)+\;5,\;\;\;\;\;\;\;x_{0}\;=\;-1.0,$$

$$f\;{\;}_{7}\;\left(x\right)\;=\;sin^{2}\;\;\left(x\right)\;-\;x{\;}^{2}\;+\;1,\;\;x_{0}\;=\;-2.5,$$

The performance of all the mention sixteenth order methods is illustrated by the following table.

Tables 3.5 illustrate a detailed comparison of the accuracy and efficiency of the proposed method against other existing sixteenth order methods for given eight nonlinear equations. The results clearly demonstrate that the proposed method is convergent to evaluate the root of all the given nonlinear equations, while some methods are divergent for some problems. The tri step algorithm (TSI) is slightly better than the proposed method but for (TSI), the Author used six number of function and the efficiency of (TSI) is $16^{1/6}\approx 1.5874$ while the efficiency of Algorithm 2.1 is $16^{1/5}\approx 1.7411$, which make the proposed method more efficient as compare to (TSI).

**Table 3.5:** The numerical experiments for Example 3.5.

Methods	Iterations	$x_{n}$	$|f(x_{{}_{n}})|$
$\;f\;{\;}_{1}\left(x\right),\;\;x_{0}\;=\;-1$			
GE1	7	-0.45662170456763682443769	4.57e-214
GE2-	2	-0.45662170156763682443769	1.36e-236
THU	5	-0.45662170156763682443769	5.68e-516
RAF	2	-0.45662170156763682443769	3.50e-320
TSI	2	-0.45662170156763682443769	2.87e-335
Algorithm 2.1	2	0.45662170156763682443769	5.67e-280
$f\;{\;}_{2}\;\left(x\right)\;,x_{0}=\;-1.2\;$			
GE1	7	-1.20764782713091892700941	1.49e-244
GE2	2	-1.20764782713091892700941	2.32e-338
THU	4	-1.20764782713091892700941	7.44e-26
RAF	2	-1.20764782713091892700941	7.66e-366
TSI	2	-1.20764782713091892700941	1.68e-521
Algorithm 2.1	2	-1.20764782713091892700941	8.9e-491
$f\;{\;}_{3}\;\left(x\right)\;,\;\;x_{0}=\;-0.9\;$			
GE1	Divergent	Divergent	Divergent
GE2	27	-0.44285440100238885381327	5.30e-1078
THU	8	-0.44285440100238885381413	8.04e-238
RAF	3	-0.44285440100238885381413	1.00e-1464
TSI	3	-0.44285440100238885381413	4.95e-2216
Algorithm 2.1	3	-0.44285440100238885381413	8.9e-1231
$f\;{\;}_{4}\;\left(x\right)\;,\;x_{0}=\;-0.9\;$			
GE1	Divergent	Divergent	Divergent
THU	6	2.69064744892861375035078	2.43e-678
RAF	4	2.69064744892861375035078	1.00e-812
TSI	3	2.69064744892861375035078	2.00e-1897
Algorithm 2.1	3	2.69064744892861375035078	8.55e-658
$f\;{\;}_{5}\;\left(x\right)\;,\;x_{0}=\;1.7\;$			
GE1	9	0.73908513321516064165531	1.29e-381
GE2	3	0.73908513321516064165531	1.79e-1969
THU	15	0.73908513321516064165531	1.65e-558
RAF	3	0.73908513321516064165531	5.09e-2574
TSI	2	0.73908513321516064165531	1.99e-242
Algorithm 2.1	2	0.73908513321516064165531	4.75e-165
$f\;{\;}_{6}\;\left(x\right)\;,\;x_{0}=\;1.7\;$			
GE1	Divergent	Divergent	Divergent
GE2	Divergent	Divergent	Divergent
THU	6	-1.20764782713091892700941	2.16e-322
RAF	Divergent	Divergent	Divergent
TSI	3	-1.20764782713091892700941	1.81e-2475
Algorithm 2.1	3	-1.20764782713091892700941	2.05e-1869
$f\;{\;}_{7}\;\left(x\right)\;,\;x_{0}=\;-2.5\;$			
GE1	17	-1.40449164821534122603506	4.58e-207
GE2	3	-1.40449164821534122603506	2.91e-856
THU	6	-1.40449164821534122603506	4.07e-582
RAF	2	-1.40449164821534122603506	2.36e-1116
TSI	3	-1.40449164821534122603506	2.06e-1755
Algorithm 2.1	4	-1.40449164821534122603506	4.00e-1080

In all the above problems the initial guess is sufficient closed to the exact root, the method is converges and determine the more reliable approximate roots as compare to the other mention methods. But in some problems due to the poor initial guess it may be slow or even diverge.

Example 3.6 [40]. Consider the nonlinear equation

x+sin(x)=0,

the initial guesses $x_{0}=1.91\;$and $x_{0}=-3.11$are poor initial guess for Newton Raphson method see [40]. For both value it’s requiring hundreds of iterations to achieve high accuracy (see Table 3.6).

**Table 3.6:** The numerical experiments for Example 3.6.

Method	Initial guess	Iteration number	$|f(x_{n})|$
Newton’s method	$x_{0}$= 1.91	450	3.0 × 10⁻¹⁹
Newton’s method	$x_{0}$= -3.11	532	-6.1 × 10⁻¹⁵
Ancient Chinese algorithm	(-3.11, 1.91)	6	2.0 × 10⁻²⁵
Bubbfil algorithm-I	(-3.11, 1.91)	3	-4.6 × 10⁻¹⁶
Bubbfil algorithm-II	(-3.11, 1.91)	2	7.2 × 10⁻¹²
Algorithm 2.1	$x_{0}$ = 1.91	3	2.0 ×$10^{-15}$
Algorithm 2.1	$x_{0}$*=* -3.11	diverge	infinity
Algorithm 2.1	$x_{0}$*=* −0.47917	1	1.2 × 10⁻¹⁵

In case of Algorithm 2.1, the initial guess $x_{0}$​=1.91 is not poor guess but if $x_{0}$= −3.11 the method is diverges. By using the He’s [40,41] approach we solve this problem. See Table 3.6.

The author of [40,41] does not use the Newton method directly for the mentioned poor initial guess and uses the Ancient Chines algorithm for the first iteration i.e $x_{1}=-0.47917$ and take this value as initial guess i.e $x_{0}=-0.47917$ for Newton method and make new Algorithms say it Bubbfil algorithm-I and Bubbfil algorithm-II.

The Algorithm 2.1 is stable if *x₀* = 1.91 and give the approximate root in only three iteration. But for *x₀* = -3.11 the Algorithm 2.1 is diverge. As for both Bubbfil algorithms if we suggest $x_{0}=-0.47917$, Algorithm 2.1 is very fast and in first iteration we obtained the approximate solution of given nonlinear equation.

## Comparison with old babylonian algorithm [41]

The Samarian used the old Babylonian algorithm to calculate the square root of non-perfect square numbers.

In modern mathematics the old Babylonian algorithm extended to solve the mth root of any number and some modern problems. In simple case, consider the cube root of a=2, the third iteration is 1.25993349 and the relative error is 0.00098%, by using the modified old Babylonian algorithm,

$$x_{n+1}=\frac{2}{3}x_{n}+\frac{a}{3x_{n}^{2}}.$$

To evaluate the cube root of 2 by using the proposed Algorithm 2.1 the relative error is very small and only 0.0000009% for the 1^st^ iteration.

## Limitations

Like the all iterative methods for the implementation of Algorthm 2.1 have some of the following limitations,

(i) f(x) must be continuous and differentiable in the neighborhood of p.
(ii) If $f^{\prime }\left(p\right)\approx 0$ the Alg 2.1 mostly failed.
(iii) If p is the repeated root of f(x)=0 the convergence rate of the of Alg 2.1 become slow or may be diverge from p.
(iv) The method is slow or may be diverge for the nonlinear equations those with sharp oscillations or closely spaced roots.

## Conclusion

In this paper, we have introduced and analyzed a highly efficient iterative method for solving nonlinear equations. A detailed convergence analysis of the proposed method is presented, demonstrating that it achieves a remarkable sixteenth-order convergence rate. Unlike many high-order methods, the proposed approach requires only first-order derivatives, making it computationally more efficient and broadly applicable compared to existing methods. This advancement provides a framework for enhancing the convergence order of optimal families of iterative methods for solving nonlinear equations. Future research should focus on developing new higher-order derivative-free methods, particularly for addressing multiple roots of nonlinear equations, sharp oscillations or closely spaced roots to further expand the applicability and efficiency of such techniques.

## Ethics statements

Not applicable.

## CRediT authorship contribution statement

**Muhammad Usman:** Methodology, Writing – review & editing. **Javed Iqbal:** Conceptualization, Methodology, Supervision. **Alamgir Khan:** Writing – original draft. **Ikram Ullah:** Investigation. **Hasib Khan:** Investigation, Validation. **Jehad Alzabut:** Methodology, Resources. **Hisham Mohammad Alkhawar:** Investigation, Validation.

## Declaration of competing interest

The authors declare that they have no known competing financial interests or personal relationships that could have appeared to influence the work reported in this paper.

## Data Availability

No data was used for the research described in the article.
